# Visual Cues Given by Humans Are Not Sufficient for Asian Elephants (*Elephas maximus*) to Find Hidden Food

**DOI:** 10.1371/journal.pone.0061174

**Published:** 2013-04-17

**Authors:** Joshua M. Plotnik, Jennifer J. Pokorny, Titiporn Keratimanochaya, Christine Webb, Hana F. Beronja, Alice Hennessy, James Hill, Virginia J. Hill, Rebecca Kiss, Caitlin Maguire, Beckett L. Melville, Violet M. B. Morrison, Dannah Seecoomar, Benjamin Singer, Jehona Ukehaxhaj, Sophia K. Vlahakis, Dora Ylli, Nicola S. Clayton, John Roberts, Emilie L. Fure, Alicia P. Duchatelier, David Getz

**Affiliations:** 1 Department of Psychology, University of Cambridge, Cambridge, United Kingdom; 2 Think Elephants International, Stone Ridge, New York, United States of America; 3 Golden Triangle Asian Elephant Foundation, Chiang Saen, Chiang Rai, Thailand; 4 Center for Mind and Brain, University of California Davis, Davis, California, United States of America; 5 Department of Psychology, Columbia University, New York, New York, United States of America; 6 M.S. 114, East Side Middle School, New York, New York, United States of America; University of Rennes 1, France

## Abstract

Recent research suggests that domesticated species – due to artificial selection by humans for specific, preferred behavioral traits – are better than wild animals at responding to visual cues given by humans about the location of hidden food. \Although this seems to be supported by studies on a range of domesticated (including dogs, goats and horses) and wild (including wolves and chimpanzees) animals, there is also evidence that exposure to humans positively influences the ability of both wild and domesticated animals to follow these same cues. Here, we test the performance of Asian elephants (*Elephas maximus*) on an object choice task that provides them with visual-only cues given by humans about the location of hidden food. Captive elephants are interesting candidates for investigating how both domestication and human exposure may impact cue-following as they represent a non-domesticated species with almost constant human interaction. As a group, the elephants (n = 7) in our study were unable to follow pointing, body orientation or a combination of both as honest signals of food location. They were, however, able to follow vocal commands with which they were already familiar in a novel context, suggesting the elephants are able to follow cues if they are sufficiently salient. Although the elephants’ inability to follow the visual cues provides partial support for the domestication hypothesis, an alternative explanation is that elephants may rely more heavily on other sensory modalities, specifically olfaction and audition. Further research will be needed to rule out this alternative explanation.

## Introduction

Although humans can easily interpret visual cues given by other humans (e.g., [1]–[3]) as informative signals [4], a growing body of research suggests that certain other animal species are also able to read human-given cues, and that such cue-following may have unique evolutionary underpinnings. The most prolific experiment to test for this ability is the object-choice paradigm, in which food or another reward is hidden under or behind one of two opaque objects. The animal subject is allowed to choose one (and only one) of the two objects after the experimenter provides the relevant cue as to the reward’s location (e.g., [5]–[7]). Object-choice experiments have been conducted on a number of species using a variety of human-initiated visual cues – including pointing, orienting and gazing – to indicate the location of hidden food.

Research findings have been mixed among the primates, particularly with regards to the great apes (e.g., [7]–[12]). Contradictory results may be due to differences in specific methodology as well as whether each cue, or a combination of cues, is examined [7], [13]–[15]. However, even individuals of the same species within the same study can show a great deal of variability [14], [16], [17]. For instance, of 12 chimpanzees tested on a gaze and point cue to find hidden food, only three individuals were able to use this information significantly above chance [16]. This variability may be explained through differences in their experience, particularly early rearing experience, with humans [17], [18], or through learning or training [19]. In fact, the ape subjects in a study by Itakura & Tanaka [10] had a great deal of human exposure and demonstrated immediate and near perfect performance. Any positive findings in monkeys have also typically only been found for a few individuals and after many trials or explicit training (capuchins (*Cebus apella*): [20]; tamarins (*Saguinus oedipus*): [21]). Exposure to humans doesn’t necessarily explain all the primate success, as there are a few cases, such as in gorillas (*Gorilla gorilla*) [11] and orangutans (*Pongo pygmaeus*) [22] where individuals who were not considered to be enculturated were able to use some human cues to find hidden food.

The mixed primate findings stand in stark contrast to studies demonstrating that domestic dogs (*Canis familiaris*) appear to spontaneously follow human cues upon initial exposure [5], [23]–[25]. One could argue that this may be due to experience with humans [5] as domestic dogs are typically raised in human environments and they perform better in comparison to wolves (*Canis lupus*) without extensive experience with humans [23], [26], [27]. That said, when Hare et al. [26] compared the performance of domestic puppies raised with littermates and puppies raised in human homes, no difference was found in their ability to follow human cues. The authors suggest that it is most likely the domestication process and not simply canid behavioral flexibility for reading visual, social cues, or differences in individual prior experience with humans that shapes the dog’s abilities for reading heterospecific social cues [26]. Although the domestication hypothesis does not preclude the fact that some non-domesticated species (especially those that are evolutionarily distant from one another) will show a capacity for so-called cooperative, visual cue-reading in an object-choice task (e.g., [10], [28]–[30]), it does suggest that in certain species, this capacity for reading human-provided visual, social cues about hidden food may have developed at some point in the domestication process as a result of man’s selection for specific social and communicative skills [24], [26]. Other domesticated species (or those closely related to domesticated species like dingoes (*Canis dingo -* [31]) that have been tested, such as horses (*Equus caballus*) [32], [33], cats (*Felis catus*) [34], goats (*Capra hircus)* [35], and foxes (*Vulpes vulpes)* bred for tameness [36] have provided further support for this hypothesis.

Experience with humans as a reason for success in these object choice tasks should not be ruled out, however, as wolves who are socialized in a similar manner to dogs may be able to learn and follow some of these cues [27], [37], [38] or may do so after extensive training [27]. Domestication also does not explain other successes among non-domesticated species, such as South African fur seals (*Arctocephalus pusillus*) [39], dolphins (*Tursiops truncatus*) [40], [41], jackdaws (*Corvus monedula*) [30], Clark’s nutcrackers (*Nucifraga columbiana*) [42], and African gray parrots (*Psittacus erithacus*) [29]. In some of these cases, the individuals tested have in fact had daily experience with humans either through training for and participation in public shows [39]–[41] or through early rearing or socialization [29], [42].

Domestication likely has allowed species such as dogs to develop the unique ability to use human cues, perhaps by an early selection to attend to a human’s face [37], but these abilities can be acquired in other ways, primarily through human experience. It is important to note, however, that in many comparative studies, results are interpreted differently by species. In ape studies, for instance, individual variability and only a single individual successfully following a visual cue may be reported as a failure, while in other species this may be reported as a success. The literature seems to hold primates to a different standard than other species, requiring group, not individual, performance above chance. This may be due to the large body of literature focused on these species as compared to non-primates.

The Asian elephant presents a unique case for examining the roles of domestication and experience because of the elephant’s long history as a beast of burden in Asia. The elephant is in fact one of the few animals currently working alongside man without any history of domestication or artificial selection for specific social traits [43]. Although the Asian elephant thus remains a genetically wild animal, its size, relative docility, and well-known social capabilities (e.g., [44]–[47]) have made it an ideal work animal for forest logging and farming for thousands of years, and for tourism more recently [43]. Asian elephants in Thailand have been used in both the logging and tourism industries for hundreds of years, often being trained for such purposes as early as 2–3 years of age and working well into senescence (60+ years of age). Captive elephants each have their own mahout (caretaker) who is responsible for their daily care, and who regularly spends 10 hours, every day with their charge. Thus, these elephants are ideal candidates for testing both human exposure and domestication as hypotheses for success in an object-choice task.

We tested seven Asian elephants at an elephant facility in Thailand on a two-object single choice task with three visual, social cues (Point, Orient, and Point&Orient). The elephants were presented with two buckets of which only one contained food. Controlling for all sensory cues except for the visual, social cue provided by the human experimenter in a given session, we tested whether or not the elephants could choose the correctly baited bucket in a significant number of trials across each social cue condition. If the results were significant and the elephants were able to use the visual, social cue to find the hidden food, this would provide evidence that human exposure underlies success in these tasks (i.e., the human-exposure hypothesis – [26]), while negative results might provide support for the domestication hypothesis. Because this experiment focused on the elephants’ ability to use only visual information provided by a human experimenter to locate hidden food, we also ran a condition with a non-visual, vocal-only command. Through prior training, the elephants were already familiar with the vocal commands given (e.g., “left,” “right,” “turn”) but in a different, non-food and non-experimental context. This test was run as a control after the visual condition had been completed for each elephant to investigate whether or not the experimental setup/apparatus was satisfactory in general for looking at the use of sensory information to find hidden food, while ensuring the visual cue testing was not confounded by any results from or learning during the vocal command condition.

## Methods

### Subjects

We tested 7 elephants (M = 2, F = 5), ranging in age from 3–42 years old, at our research facility at the Golden Triangle Asian Elephant Foundation (GTAEF) in Chiang Saen, Thailand. The facility is home to 30 elephants, some of which are rented (and rescued from the streets of Bangkok) as part of the elephant camp programs at the Anantara Golden Triangle Resort and Spa and the Four Seasons Golden Triangle Tented Camp. The elephant’s mahout (the daily caretaker who is also usually the elephant’s owner), two full-time staff veterinarians, and senior management provide daily care and ensure proper elephant welfare practice is in place. Five of the elephants we tested have been with their respective mahouts for at least ten years, while two under the age of 10 have been paired with their mahouts and housed at GTAEF since birth. The mahout is responsible for daily care and husbandry, and generally works with his elephant for up to 10 hours a day, seven days a week. Although the elephants have undergone extensive training to respond to a variety of visual, tactile and vocal commands that control their movement, our study employed visual commands that were never explicitly trained. Specifically, the elephants had no prior experience with either the apparatus used or training for the experimental, visual cues provided to them in this study. This study was approved by the National Research Council of Thailand, and by the University of Cambridge Zoology Animal Users Committee (Z003/2011).

### Apparatus – Sliding Table

**Figure 1:**
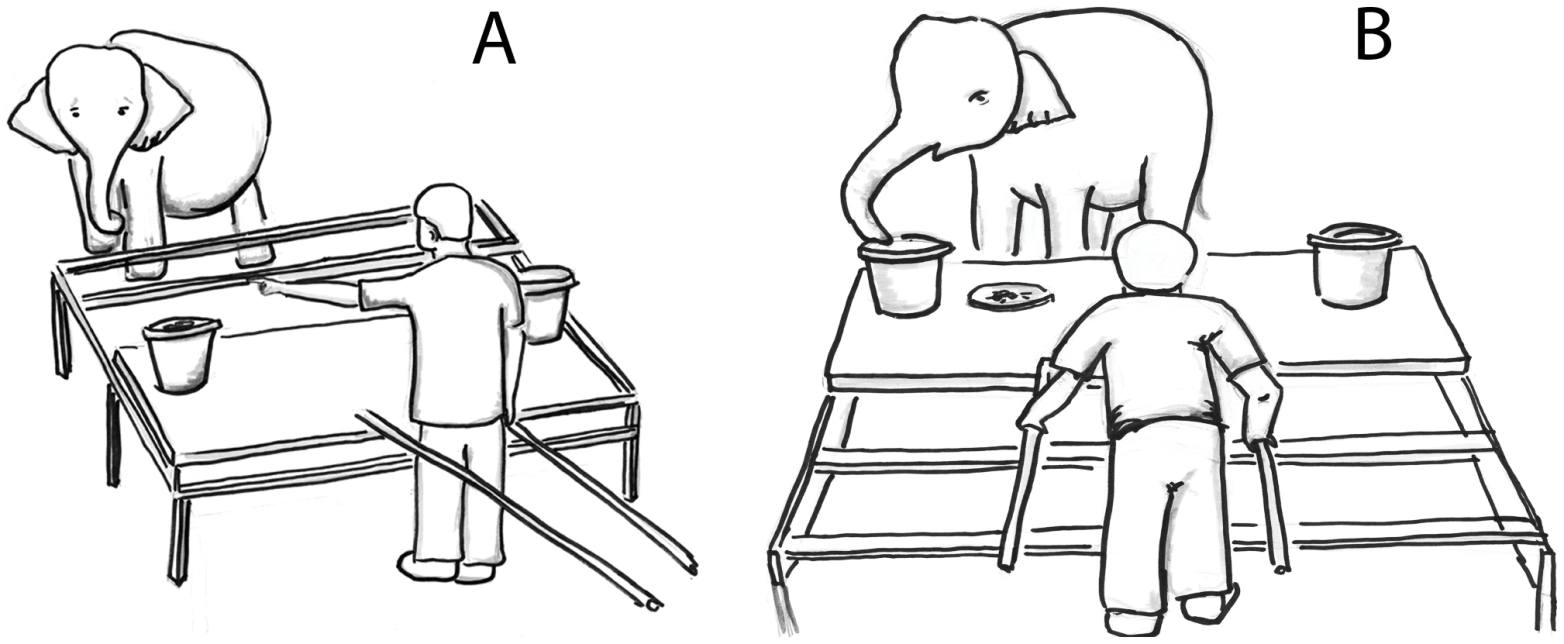
Diagrams of apparatus and elephant testing. A) The experimenter gives the “point” cue to the bucket to the elephant’s right. B). The elephant makes the correct choice after the table is pushed forward. Drawings by A. Hennessy.

A sliding platform was used to quickly extend and retract baited buckets toward and away from the subject as shown in Figure 1. The platform, measuring 2.97×0.90 meters, was fitted with wheels that rolled within grooves on a support frame. The square frame measured 3 meters along each side and stood 0.54 meters off of the ground. With the platform resting on top of the frame, the entire structure reached a height of 0.67 meters. Attached to the rear of the platform were two cylindrical arms (2.02 m), which served as push/pull handles. A curtain (length = 4.66 m, height 2.77 m) was rigged up on a pulley system, a distance of 1.24 meters to the front of the platform frame. Two green plastic buckets with lids placed upside down were positioned 2.46 m apart on the sliding table.

### General Procedure

At the beginning of each trial, the elephant waited behind the curtain – the mahout was present at all times but instructed to remain behind the elephant and refrain from giving any other cues - so that the elephant had no premature visual access to the experimental apparatus. The curtain was drawn while the buckets were baited. Two experimenters – the first author (J.M.P.) and the elephant’s mahout - were present at all times, one (A) at the table, the other (B) at the curtain rope. Experimenter A baited a bucket and positioned himself between the two buckets and behind the table. After experimenter A gave a single word cue, experimenter B opened the curtain and the elephant was allowed to approach the table. If the elephant did not approach the table, the elephant’s name was repeated with the single Thai command “ma”, or come. As soon as the elephant approached the table, experimenter A provided one of three single or combined visual cues to the elephant for five seconds. The cue was then withdrawn, and the table pushed toward the elephant by the forward-facing experimenter. The elephant was allowed to make a single choice by sliding the lid off the chosen bucket and, if correct, eating the food – a handful of sunflower seeds – inside. As soon as the elephant removed their trunk from the bucket whether or not they had made the correct choice, or when they attempted to move from an incorrectly chosen bucket to the correct one, the table was pulled back and the curtain drawn.

### Visual Cue Procedure

Prior to the beginning of test trials for each elephant, subjects were presented with two pre-testing conditions: 1) warm-up trials: a minimum of 6 trials in which one of the buckets was baited in view of the elephants with the curtain open, and 2) 4 baseline trials, which were identical to control trials (see below). To proceed to baseline trials, elephants needed to choose correctly in 4/6 warm-up trials, or in three consecutive trials thereafter.

In subsequent test trials, the curtain was drawn, and buckets were baited and sham-baited randomly to control for possible sound and olfactory cues; this was to ensure that the only cue about the hidden food available to the elephant was the visual cue provided by the experimenter. Elephants were subject to three experimental conditions – point only, orient only, and point and orient - provided by two different experimenters (J.M.P. and the elephant’s mahout). In the point only condition (Point), the experimenter faced directly ahead, toward the elephant, and pointed with the hand closest to the baited bucket using an extended index finger (Figure 1). In the orient only condition (Orient), the experimenter kept both hands at his side and turned the entire body approximately 45 degrees to face the baited bucket. In the point and orient condition (Point&Orient), the experimenter oriented as in the Orient condition, and used the contralateral hand to point toward the baited bucket. Control trials were the same as test trials except that the experimenter faced the elephant throughout the entire trial and no visual cue was given during the five-second delay prior to the table’s presentation. Each elephant was subjected to one block of 16 trials per day (12 pseudo-randomly dispersed test trials and 4 pseudo-randomly dispersed control trials, with control trials never being presented consecutively nor as any of the first four trials in a block, and no bucket being baited more than three consecutive times in a row), and two blocks of each condition, per experimenter, during the course of the experiment. The second block of each condition was repeated only after all three conditions had been run once per experimenter. No more than three elephants were presented with the same order of condition, with condition presentation counter-balanced between individuals. Each elephant was presented with 12 total blocks of trials, and 144 total test trials.

One set of 12 probe trials per experimenter was run after all experimental sets were completed to assess whether or not elephants relied on one cue over the other. In these trials, both buckets were baited, and both cues (pointing and orienting) were presented, one in each direction (e.g., point to the left bucket, orient to the right bucket).

### Vocal Command Procedure

Following completion of visual cue testing for all elephants, we ran two sets of 16 trials (12 test trials, and 4 control trials identical to those in the visual cue tests randomized as per the visual cue procedure) per experimenter on each elephant, in which elephants were presented with a vocal, and no visual command. The order of condition presentation was counterbalanced between elephants. In test trials, the experimenter stood facing the elephant, and directed the elephant to the correct bucket by providing only either a directional vocal cue (the Thai word for “left” or “right” – n = 4), or a non-directional vocal cue (the Thai word for “turn” – n = 3) for five seconds prior to the presentation of the table. The cue given was dependent on the training experience of the elephant.

### Analyses

We used Heterogeneity G-tests to test for side biases across the elephants for each experimenter, and to test group performance on each condition of, and with each experimenter on the object choice task. These tests compare performance with random chance, but unlike the chi-square, they take both individual contributions and the directionality (heterogeneity) of the data into account. We used the binomial test to compare each individual elephant’s performance on each condition to chance. We used a Friedman test to assess whether the elephants performed better for any of the three visual conditions, and Paired-Sample Wilcoxon Signed Ranks Tests to compare the elephants’ performance between the two experimenters, and to compare vocal command test and control trials [48].

## Results

### Visual Cues

All of the elephants reached criterion in the pre-test, warm-up trials (mean number of trials to criterion = 8.14). In test trials, because the elephant was positioned in front of the sliding apparatus so that one bucket was on either side of its body when it made a choice, we first assessed whether the subjects had a side bias to the left (L) or the right (R) bucket, regardless of whether it was the correct choice, by performing Heterogeneity G-tests on the main effects of experimenter and condition type using number of L responses for each subject. Results were significant for both experimenters (J.M.P. and the mahout) when condition type was combined (J.M.P: Gh = 57.60, df = 6, P<0.01, Gt = 57.61, d = 7, P<0.01; Mahout: Gh = 35.45, df = 6, P<0.01, Gt = 37.47, d = 7, P<0.01), indicating that as a group there was a significant side bias demonstrated by the elephants with both experimenters. Gh, a measure of the heterogeneity of the data, was significant due to the group not having a consistent side bias (e.g., some subjects were biased toward the right while others were biased toward the left). Assessing L/R responses to each condition irrespective of the experimenter also resulted in a significant side bias in the Point&Orient (Gh = 40.38, df = 6, P<0.01, Gt = 43.07, d = 7, P<0.01) and Orient (Gh = 42.56, df = 6, P<0.01, Gt = 42.57, d = 7, P<0.01) conditions, but not in the Point condition (Gh = 10.37, df = 6, p = n.s., Gt = 10.38, d = 7, P = n.s.). Because of the heterogeneity of the data, we would need to split the elephants into two groups based on their specific preferred choice (L or R) to analyze the side bias data by individual across sessions. We were unable to do this because it would reduce the sample size of each group to less than 5, a number too small for statistical analysis.

To assess whether the visual cue given (Point, Orient or Point&Orient) had any significant effect on the elephant’s bucket choice, we performed a Friedman Test on accuracy data across all (N = 7) elephants. There was no significant difference between the three visual cue conditions (Point Median = 24.0, Orient Median = 24.0, Point&Orient Median = 24.0; χ^2^_2_ = 2.08, P = n.s.). A Wilcoxon Signed Ranks Test showed that the experimenter giving the visual cue also did not affect the performance accuracy of the elephants (J.M.P Median = 38.0, Mahout Median = 34.0; T^+^ = 23.5, T^−^ = 4.5, N = 7, P = n.s.), although there was a trend toward the elephants’ performing better for J.M.P. (P = 0.078). The group did not perform significantly above chance on any condition (Heterogeneity G-test, Point: Gh = 8.40, df = 6, P = n.s., Gp = 1.44, df = 1, P = n.s.; Orient: Gh = 7.39, df = 6, P = n.s., Gp = 0.05, df = 1, P = n.s.; Point&Orient: Gh = 3.15, df = 6, P = n.s., Gp = 0.11, df = 1, P = n.s.) or with either experimenter (Heterogeneity G-test, J.M.P.: Gh = 4.65, df = 6, P = n.s., Gp = 1.79, df = 1, P = n.s.; Mahout: Gh = 11.21, df = 6, P = n.s., Gp = 0.20, df = 1, P = n.s.).

While as a group the elephants did not perform significantly above chance on any condition, we did look at individual performances given our small sample size. Subjects needed to get at least 17 out of 24 trials correct on any condition to be significantly above chance (binomial test, α = 0.05, P<0.05). Only one of the seven elephants performed above chance (17/24 correct), and only in one of the three conditions (Table 1).

**Table 1:** Number of correct trials by each subject, in each visual cue condition –“Point,” Point&Orient (“P&O”), and “Orient”– and their respective controls (“Control”), probe condition (“Probe”), and vocal command condition (“Vocal”) and their respective controls (“VControl”).

Subject	Point JMP	Point M	P&O JMP	P&O M	Orient JMP	Orient JMP	Probe JMP	ProbeM	Control JMP	ControlM	Vocal JMP	VocalM	VControl JMP	VControl M
Ploy	17*	17*	12	14	16	16	7	8	8	12	22*	23*	4	4
Bo	14	10	9	15	12	9	4	4	9	13	21*	15	5	3
Am	13	10	9	12	12	13	3	5	14	11	20*	20*	5	3
Puki	16	11	12	12	10	11	6	6	11	12	19*	14	5	4
NamFon	14	11	15	12	11	14	6	7	12	8	21*	18*	4	2
Pepsi	12	12	14	9	13	9	6	3	12	9	19*	16	4	4
TangMo	13	9	11	9	12	12	8	8	13	11	23*	18*	3	6
*Mean*	14.1	11.4	11.7	11.9	12.3	12	5.7	5.9	11.3	10.9	20.7	17.7	4.3	3.7

Columns are delineated by cue given and experimenter (J.M.P. is the first author, M is the elephant’s mahout). Each cell is out of 24 trials except Probe and VControl cells, which are out of 12 and 8 trials respectively.

* P<0.05 for binomial test.

In probe trials where the two experimenters (J.M.P. and the elephant’s mahout) gave the point cue to one bucket and the orient cue to the other, elephants did not choose significantly more often based on either cue (Heterogeneity G-test, J.M.P.: Gh = 6.00, df = 6, P = n.s., Gp = 0.19, df = 1, P = n.s., Mahout: Gh = 7.84, df = 6, P = n.s., Gp = 0.05, df = 1, P = n.s.).

### Vocal Command

Table 1 shows results of binomial tests on the elephants’ performance in 24 trials per experimenter across the vocal command condition. All of the elephants performed significantly better than chance on trials in which J.M.P. gave the vocal command, while 4 of 7 elephants did so when it was the mahout giving the command. This was confirmed with a group-level analysis of success compared to chance performance which showed that the elephants were able to follow the vocal command to find the location of hidden food when both J.M.P. (Median = 21.0; Gh = 5.16, df = 6, P = n.s., Gp = 98.73, df = 1, P<0.01) and the mahout (Median = 18.0; Gh = 14.38, df = 6, P = 0.03, Gp = 39.68, df = 1, P<0.01) gave the command. The elephants performed significantly better when J.M.P. was the experimenter giving the vocal command rather than the mahout (Wilcoxon Signed Ranks Test: T^+^ = 20.0, T^−^ = 1.0, N = 6, P = 0.031).

## Discussion

As a group, the elephants did not use either of the two individual visual, social cues or the combined visual cue to locate the hidden food. In addition, in probe trials where the elephants were given two, opposing cues toward each of two baited buckets, the elephants failed to follow any one cue over another. This result suggests that even in later trials, the elephants were not using any specific, visual information to locate the hidden food. The elephants did successfully use the vocal command cue to locate the hidden food in the object choice task, however, suggesting that they were able to use human-provided cues to find the food if the cues were sufficiently salient (see also studies that show apes do better if adding vocalizations to the object choice task [17], [49]). Also, the elephants performed better with J.M.P. as the experimenter than the mahout on the vocal command condition, which may suggest that the relationship between the individual giving the cue and the elephant may affect their performance.

The data suggest that as a group, the elephants had an overwhelming side bias in their performance on the object choice task, and thus many of the elephants had a preference for selecting either the left or the right bucket during testing. If the elephants genuinely have a strong side bias to one side, then the non-significant results on the visual cue tests may be a result of this side bias and not their inability to follow the visual cues; in other words, adopting a strong side bias may obscure our ability to detect whether or not the animals can use visual cues. An alternative explanation is that the elephants were unable to use the visual cue provided by the experimenter to find the food and as a consequence, they adopted a “choose one bucket and stick to it” strategy for maximizing their food intake. The first explanation seems unlikely given their success on the vocal commands (meaning they chose the correctly baited bucket in a significant number of trials regardless of the bucket’s location), and their ability to follow the placement of the food in the pre-test, warm-up trials. So, even if the elephants had a side bias, they were able to use specific cues (either seeing the food or hearing a command) to overcome them. In addition, we continued to use the experimental apparatus for other, later cognitive tasks in which the elephants never showed a side bias. After approximately two months of such testing, we ran two additional sets of the “Point” condition; the elephants performed just as poorly as they had in original tests. The second explanation thus seems to be the more likely reason for the elephants’ side biases. This is a rather common phenomenon in animal cognition tasks where animals fail and thus try to maximize perceived success [50].

Our results suggest support for the domestication hypothesis in the sense that the Asian elephants, a non-domesticated species, did not follow the visual cues provided by the experimenters to find food. But these results are also interesting in light of an informal survey we ran on the mahouts. All of the seven mahouts in this study had worked on a daily basis with their elephant charge for years. When asked whether or not their elephants followed pointing cues, the mahouts responded that they used such cues on a daily basis to instruct their elephants to pick up specific objects. In particular, the mahouts noted that they often asked their elephant – using the pointing cue - to pick up a tourist’s flip flops or hat from the ground and to hand these objects to the tourist sitting on the elephant’s back. The mahouts were in fact visibly frustrated when their elephants failed to read either J.M.P.’s or their own cues during the experiment (which perhaps affected the results and explains why the elephants performed slightly better when J.M.P. was the experimenter), so we implemented the vocal command cue as a control to see whether or not the elephants’ performance on the visual task was a result of their inability to read the cue or perhaps a problem with the apparatus and experimental setting. These results suggested that the elephants were capable of following other, non-visual cues with which they had direct, prior experience. Although the elephants had significant experience with the vocal command cue (particularly at non-testing times when the mahout was sitting on the elephant’s back or neck giving directional commands vocally and tactilely), they theoretically should have had relatively equal experience with visual commands or cues as well (when the mahout was on the ground giving directional commands with a combination of cues). Because we cannot be sure of this, we did not compare the elephants’ results across the two modalities. However, their ability to use the vocal but not the visual cue in a novel situation suggests that the elephants’ ability to solve problems may rely more on non-visual sensory information, and in the case of human-given cues, on non-visual cues with which they have substantial prior experience. Thus, although the elephants failed to follow the visual cue given by humans, it is difficult for us to interpret our results in terms of the domestication hypothesis because the social cue we provided may not be sufficiently salient for the elephant. Their inability to follow the three visual, social cues provided in this experiment may be a result of elephants’ overwhelming use of olfactory and acoustic information when navigating their environment (e.g., [51]–[53]) rather than their genetic lack of “domesticated” social traits. Interestingly, when observing the mahouts interacting with their elephants, it appears likely that it is the mahout’s overall behavior (i.e., their body orientation’s effect on the transmission of their vocal command and the direction toward which the elephant is facing), not to mention the smell of the object or food item the mahout is requesting, that helps the elephant successfully follow the command. Although captive elephants’ longtime human exposure may play an important role in the elephants’ following of specific cues provided by their mahouts, perhaps the sensory modality in which the cue is read is particularly relevant.

Our previous work on mirror self-recognition in elephants suggested they might make good candidates for studies that provide visual cues about hidden food [[54], J. Plotnik, personal observation], and thus a good comparison species in tests of the domestication hypothesis. However, the mirror experiments provide the animals with a clear, substantial and unwavering image that is easily manipulated and tested by the animal itself, for a relatively prolonged period of time. The traditional visual cue study, including the current one, provides a human-given, subtle gesture that may differ in its salience from species to species. Thus, although elephants can see well enough to follow the mirror reflection, this study’s results suggest they may use other sensory systems in physical or social contexts such as problem-solving, foraging or communication.

The ability to compare species across evolutionary taxa using similar research questions and relatively similar experimental apparatuses requires that each individual species’ sensory and problem-solving capacities are assessed and acknowledged. Before this is accomplished, the ability to compare these vastly different species (including primates, cetaceans, canids, birds, and elephants) and interpret the resulting data is limited. Significant studies on elephants’ sensory abilities beyond the visual domain will provide a clearer picture of how they “see” their natural world, and will help to better inform studies of convergent cognitive evolution.

Here, we have demonstrated that in an object choice task the elephants do not use three widely used (both in the elephant’s daily life and across empirical studies on other animals) visual, social cues given by a human experimenter to find hidden food, but can follow a human’s vocal commands on which they had previously been trained in a completely different context. Elephants are well-known for their social complexity and communicative abilities (e.g., [44]–[46], [51]–[53], [55]–[58]) which suggests that elephants’ ability to problem-solve using social information may be comparable to other species. This ability, however, may rely on non-visual sensory information, which makes the elephant an interesting subject for studying how non-primates “view” their natural world.
